# Epidemiology and associated factors of gingivitis in adolescents in Guangdong Province, Southern China: a cross-sectional study

**DOI:** 10.1186/s12903-021-01666-1

**Published:** 2021-06-16

**Authors:** Weihua Fan, Conghua Liu, Yazhi Zhang, Zijing Yang, Jianbo Li, Shaohong Huang

**Affiliations:** 1 Stomatological Hospital, Southern Medical University and Guangdong Provincial Stomatological Hospital, 366 Jiangnan Avenue South, Guangzhou, 510260 China; 2 Department of Biostatistics, School of Public Health (Guangdong Provincial Key Laboratory of Tropical Disease Research), Southern Medical University, Guangzhou, 510515 China

**Keywords:** Gingivitis, Dental calculus, Adolescence, WHO Community Periodontal Index probe

## Abstract

**Background:**

Gingivitis is the most prevalent form of periodontal disease in children and adolescents, being strongly associated to some socioeconomic factors and oral health behaviours. This study aimed to assess the prevalence of gingivitis and its association with socio-demographic factors and oral health-related behaviours in children aged 12–15 years in Guangdong, Southern China.

**Methods:**

A total of 7680 children were sampled using an equal-sized, stratified, multistage, random sampling method and clinically examined between December 2015 and April 2016. A questionnaire on socio-demographic factors and oral health-related behaviours related to gingivitis was completed by each of the selected children. Gingival bleeding was recorded using the Community Periodontal Index probe, and children with a gingival bleeding positive score ≥ 10% were defined as having gingivitis. A multivariate logistic regression analysis was performed to assess the association between socio-demographic factors and gingivitis. All statistical tests were performed at a two-sided significance level of 0.05.

**Results:**

The weighted prevalence of gingivitis among 12–15-year-old children was 29.6%, with 22.6% having localised gingivitis and 7.0% having generalised gingivitis. Age differences were observed in the prevalence of gingivitis, whereas urban-rural differences were not. According to the multivariate logistic regression analysis results, factors such as increasing age, being the only child, lack of regular annual dental check-up, and heavy dental calculus were significantly associated with higher prevalence of gingivitis. In addition, the association of gingivitis with these factors was inconsistent among the urban and rural areas.

**Conclusions:**

Dental calculus and oral health behaviour were found to be important factors for maintaining the gingival health of children aged 12–15 years in Guangdong. Maintaining gingival health in children requires promoting positive oral health behaviours and regular dental prophylaxis.

## Background

Gingivitis is characterised by gingival redness, swelling, bleeding, and the absence of periodontal attachment loss (AL) [1].  It is commonly painless and rarely leads to spontaneous bleeding, with most patients being unaware of the disease or unable to recognise it [2]. Plaque-induced gingivitis is the most prevalent form of periodontal disease in children and adolescents [3–5]. Most children exhibit signs and symptoms of gingivitis [6]. The prevalence of gingivitis ranges from 23 to 77% in young Latin American individuals [7].

A large number of epidemiological studies assessed the prevalence of periodontal diseases using the Community Periodontal Index (CPI) of Treatment Needs (CPITN) or gingival index (GI) for evaluating gingival inflammation [8–13]. The prevalence of gingivitis was usually defined as the presence of gingival bleeding in at least one site or sextant [8–10]. However, a gingival inflammatory site or sextant does not necessarily equate to a gingivitis case or individual; thus, the CPITN is not a suitable tool for defining a gingivitis case [14, 15]. Recently, the joint EFP/AAP workshop published a new definition of gingivitis, by which it set the threshold for gingivitis as the presence of ≥ 10% of bleeding sites; thus, a patient presenting with a bleeding on probing (BOP) score < 10% without periodontal AL and radiographic bone loss (intact periodontium) is considered clinically periodontally healthy [15]. This definition is also recommended for epidemiological investigations [15].

Gingival tissue is more sensitive to plaque biofilm. Gingival inflammation may be exacerbated in young individuals during puberty. High levels of circulating sex hormones are associated with gingival inflammation [3, 16]. Other risk factors for gingivitis include oral health behaviours, gender [17], oral hygiene, dental calculus [18], and socioeconomic factors [11, 19, 20]. Good oral health in early childhood contributes to better oral health in adulthood. Chronic infections of the gingival tissues in children and adolescents may have an impact on their future oral and systemic health in adulthood [21]. Gingivitis should be identified and treated in young individuals, as persistent gingivitis represents a risk factor for the development of periodontitis [22]. Over the past 10 years, Guangdong, which is at the core of Southern China, has made progress in social development and urbanisation, and had rapid economic growth. There have been dramatic changes in population (approximately 115 million permanent residents in 2019) and lifestyle, which might have had an impact on the oral health and related behaviours of adolescents. Our study aims to enhance the monitoring of adolescent oral health status and health-related influencing factors through a better understanding of the socioeconomic factors that affect oral health and help healthcare professionals improve the oral health status of adolescents. Considering that gingival bleeding is universally accepted and recommended by the World Health Organization (WHO) as a parameter to assess gingival health status in large-sample field epidemiological surveys, we chose to assess gingival bleeding instead of BOP to assess the prevalence of gingivitis and the association of gingivitis with socio-demographic factors and oral health-related behaviours in children aged 12–15 years in Guangdong.

## Methods

### Study design, sampling, and sample sizes

The study utilised a stratified, multistage, random sampling design. School children residing in Guangdong for more than 6 months were approached to participate in this survey. Our sampling method included four age subgroups (12-, 13-, 14-, and 15-year-old). The sample size in each group *n* was calculated using the following equation: $n=Z_{{1 - \alpha /2}}^{2}p(1 - p)/{\delta ^2}$, where ${Z_{1 - \alpha /2}}$ is the $(1 - \alpha /2) \times 100\%$ quantile of the standard normal distribution. According to the Third Guangdong Provincial Oral Health Survey conducted in 2005 [23], the expected prevalence of gingivitis was *p* = 0.338 and the allowable error was *δ* = 0.05*p*. With the level of statistical significance *α* set at 0.05, an estimated sample size was 1536 persons per group. An additional 10% sampling was performed in each school to prevent non-response. The actual number of participants in each group was 1920, with 1:1 ratio of urban-to-rural and males-to-females. The study participants were recruited using an equal-sized, stratified, multistage, random sampling method with probability proportional to size. The sampling process consisted of three stages according to administrative regions: first, four districts and four counties were chosen; second, three middle schools in each district and county were chosen; and finally, in the field investigation, a total of 320 school children aged 12, 13, 14, and 15 years in each middle school were recruited using the quota sampling method. Parents and children were informed regarding the study and they signed the informed consent forms. The informed consent was in accordance with the Declaration of Helsinki and approved by the Stomatological Ethics Committee of the Chinese Stomatological Association on 9 July 2014 (Approval No.: 2014-003).

### Data collection

#### Dental examination

Field investigation was carried out between December 2015 and April 2016. Data were collected from dental examination records and structured questionnaires. Dental examination was conducted by three dental examiners in the school’s medical offices. Instruments used for oral examination were the following: metallic WHO CPI probe, plane mouth mirrors with LED light, and mobile dental chairs. All examiners were trained and calibrated by at least one experienced periodontist. The Kappa scores of inter- and intra-examinations for periodontal pocket (PD) and AL were 0.62–0.92. The criteria for periodontal examination were based on the Oral Health Surveys—Basic Methods, 5th edition (2013) [24]. Gingiva of all teeth excluding the third molars were examined. The CPI probe tip was inserted gently into the gingival sulcus or pocket and the full extent of the sulcus or pocket was explored. The probe was moved gently with short upward and downward movements following the anatomical configuration of the surface of the tooth root to assess the absence or presence of bleeding response. The sensing force used was no more than 20 g. Gingival bleeding was scored as 0 (healthy) or 1 (bleeding). An individual gingival bleeding score was assessed as the proportion of bleeding teeth to the total number of teeth examined. A child with a gingival bleeding positive score ≥ 10% without AL or PD (≥ 4 mm) was defined as having gingivitis, which was further classified as localised (gingival bleeding score ≥ 10% and ≤ 30%) or generalised (gingival bleeding score > 30%). According to the WHO criteria, several clinical conditions were also assessed, such as the prevalence of caries (including decayed, missing, and filled teeth), calculus, PDs, and AL (recorded only in the 15-year-old group). At the end of clinical examinations, participants who were diagnosed with gingivitis were provided a report of their condition and advised to seek an oral health consultation.

#### Self-assessment of oral health using questionnaires

Before the initiation of dental examinations, two trained investigators instructed the students to fill out a self-administered questionnaire. The structured questionnaire was taken from the Fourth National Oral Health Survey [25], consisting of questions on socio-demographic characteristics (age, gender, if only child, parental educational levels, area of residence, and region), oral health knowledge and attitudes (bleeding from brushing is normal, bacteria can cause gingivitis, oral diseases may affect general health, and brushing does not prevent gingivitis), and oral health behaviours (smoking, alcohol consumption, consumption of sugars and sweet foods, daily frequency of tooth brushing, dental floss use, and annual dental check-up).

### Statistical analysis

EpiData 3.0 software (The EpiData Association, Odense, Denmark) was used to input data. After entry and a logic check the data were analysed using SPSS 19.0 software (IBM SPSS, IBM Corp, Armonk, NY). The categorical data were expressed by the number of cases (percentage), and the chi-square test was performed for comparison between groups. As the distribution of gingival bleeding does not conform to normal distribution, the nonparametric test was used to compare the differences between the groups. Subsequently, an unconditional logistic model was run to screen the possible influencing factors. Only variables that generated a *P*-value ≤ 0.20 in the unadjusted analyses were considered for the model, and they were retained in the multivariate logistic regression model only if the *P*-value was < 0.05 after adjustment. Considering the influence of gender, parental educational level, dental floss use, and daily frequency of tooth brushing on the results [17], they were also included as confounding factors in the multivariate analysis. Concurrently, the total sample was divided into two sample subgroups, and logistic regression models were established for these two groups to determine whether there were differences between the regression coefficients in the urban subgroup and the rural subgroup. In order to compare the coefficients of these two regression models, we added the interaction terms between the area variable and the rest of the variables in the model and assessed differences in the coefficients by the significance of the interaction terms. In addition, considering the complex sampling and possible bias, all data were weighted by the gender and age of the permanent residents of Guangdong Province in 2010 published by the National Bureau of Statistics. All statistical tests were performed at a two-sided significance level of 0.05.

## Results

In this survey, a total of 7680 students were examined, and they completed the questionnaire. The gingival health status of adolescents is shown in Table 1. The mean number of teeth with gingival bleeding in 12–15-year-old adolescents was 2.26 ± 3.77. There were significant differences in the number of teeth with gingival bleeding among the different age groups (*Z* = 25.572, *P* < 0.001), with 1.96 ± 3.22 being the number of teeth with gingival bleeding in the 12-year-old age group; this increased to 2.40 ± 4.20 in the 15-year-old age group. The total prevalence of gingivitis among 12–15-year-old adolescents was 29.6%, with 22.6% having localised gingivitis and 7.0% having generalised gingivitis. Similar to that of the mean number of teeth with gingival bleeding, age influenced the prevalence of gingivitis (*χ*^2^ = 18.320, *P* < 0.001). In addition, the prevalence of gingivitis was higher in males than in females (30.6% vs. 28.6 %, *P* = 0.032), but there were no statistically significant differences in the prevalence of gingivitis between the urban and rural populations (*χ*^2^ = 0.999, *P* = 0.318), or between children of parents with different educational levels. In the 15-year-old group, the prevalence of periodontitis was low, with rates of PD (≥ 4 mm) and AL (≥ 4 mm) of 0.57 and 0.1%, respectively.

**Table 1:** The prevalence of gingivitis and mean numbers of teeth with gingival bleeding among 12–15 years old children. (weighted)

Variable	Category	*N*	Gingivitis^†^			Localized gingivitis^‡^			Generalized gingivitis*			Gingival bleeding teeth	
			N	%	95% CI	n	%	95% CI	n	%	95% CI	$\bar{x}$	SD
Age (year)	12	1920	523	27.2	(25.2, 29.2)	419	21.8	(20.0, 23.7)	104	5.4	(4.4, 6.4)	1.96	3.22
	13	1920	538	28.0	(26.0, 30.0)	416	21.7	(19.8, 23.5)	122	6.4	(5.3, 7.4)	2.09	3.45
	14	1920	633	33.0	(30.9, 35.0)	479	24.9	(23.0, 26.9)	154	8.0	(6.8, 9.2)	2.59	4.07
	15	1920	582	30.3	(28.3, 32.4)	424	22.1	(20.2, 23.9)	158	8.2	(7.0, 9.5)	2.40	4.20
Gender	Male	3840	1176	30.6	(29.2, 32.1)	887	23.1	(21.8, 24.4)	289	7.5	(6.7, 8.4)	2.33	3.84
	Female	3840	1100	28.6	(27.2, 30.1)	851	22.1	(20.8, 23.5)	249	6.5	(5.7, 7.3)	2.17	3.67
Area	Urban	3840	1158	30.2	(28.7, 31.6)	864	22.5	(21.2, 23.8)	294	7.7	(6.8 8.5)	2.31	3.84
	Rural	3840	1118	29.1	(27.7, 30.6)	874	22.8	(21.4, 24.1)	243	6.3	(5.6, 7.1)	2.21	3.69
Father’s Education level	≤ 9 years	4346	1309	30.1	(28.8, 31.5)	1005	23.1	(21.9, 24.4)	304	7.0	(6.2, 7.8)	2.30	3.81
	10 –12 years	1474	423	28.7	(26.4, 31.0)	310	21.0	(19.0, 23.1)	113	7.7	(6.3, 9.0)	2.27	3.93
	≥ 13 years	804	230	28.6	(25.5, 31.7)	174	21.6	(18.8, 24.5)	56	7.0	(5.2, 8.7)	2.11	3.53
	Unclear	1057	314	29.7	(27.0, 32.5)	249	23.6	(21.0, 26.1)	65	6.1	(4.7, 7.6)	2.19	3.52
Mother’s Education level	≤ 9 years	4871	1467	30.1	(28.8, 31.4)	1120	23.0	(21.8, 24.2)	346	7.1	(6.4, 7.8)	2.30	3.79
	10–12 years	1074	313	29.1	(26.4, 31.9)	230	21.4	(19.0, 23.9)	83	7.7	(6.1, 9.3)	2.26	3.88
	≥ 13 years	678	185	27.3	(23.9, 30.6)	146	21.5	(18.4, 24.6)	39	5.8	(4.0, 7.5)	1.98	3.46
	Unclear	1045	311	29.8	(27.0, 32.5)	242	23.2	(20.6, 25.7)	70	6.7	(5.2, 8.2)	2.27	3.72
Total		7680	2276	29.6	(28.6, 30.7)	1738	22.6	(21.7, 23.6)	538	7.0	(6.4, 7.6)	2.26	3.77

†Prevalence of gingivitis (calculated as a proportion of gingival bleeding score ≥ 10%)

‖: The results of the chi-square test

‡: Prevalence of localized gingivitis (calculated as a proportion of gingival bleeding score ≥ 10% and ≤ 30%)

*: Prevalence of generalized gingivitis (calculated as a proportion of gingival bleeding score > 30%)

¶: The results of the Kruskal–Wallis H test

The results of the unadjusted associations between the socio-demographic, oral health behaviour and attitudes, and oral health status variables associated with gingivitis are displayed in Table 2. Higher prevalence of gingivitis was possibly associated with the socio-demographic characteristics of older age, male sex, not being an only child, and lower levels of the mother’s education. In addition, higher prevalence of gingivitis was possibly associated with poor oral health knowledge and attitudes, such as the ‘bleeding during brushing is normal’ or ‘brushing does not prevent gingivitis’ attitude, and poor oral health behaviours, such as brushing less than twice a day, not flossing daily, smoking, not undergoing annual dental check-up, and consuming sugar or sweet foods more than once a day. An adverse oral health condition such as calculus, especially heavy calculus, was a high-risk factor for gingivitis; however, dental caries did not affect the risk for gingivitis in children.

**Table 2:** Association between socio-demographic, knowledge, behavior factors and gingivitis prevalence according to univariate logistic regression analysis

Variables	OR (95% CI)	*P* value
*Socio-demographic characteristics*		
Age (year)		0.002
12	Ref.	
13	1.040 (0.902, 1.198)	0.591
14	1.315 (1.145, 1.510)	< 0.001
15	1.160 (1.009, 1.335)	0.037
Gender		
Male	Ref.	
Female	0.898 (0.813, 0.991)	0.032
Area		
Urban	Ref.	
Rural	0.950 (0.862, 1.048)	0.309
Only child		
No	Ref.	
Yes	0.899 (0.801, 1.008)	0.068
Father’s Education level		0.248
≤ 9 years	Ref.	
10–12 years	0.934 (0.820, 1.064)	0.306
≥ 13 years	0.929 (0.786, 1.096)	0.382
Unclear	0.979 (0.845. 1.134)	0.776
Mother’s Education level		0.095
≤ 9 years	Ref.	
10–12 years	0.939 (0.812, 1.085)	0.392
≥ 13 years	0.868 (0.725, 1.039)	0.123
Unclear	0.985 (0.851, 1.140)	0.839
*Oral health knowledge*		
Bleeding from brushing is normal		
Incorrect answer	Ref.	
Correct answer	0.892 (0.802, 0.992)	0.035
Bacteria can cause gingivitis		
Incorrect answer	Ref.	
Correct answer	1.036 (0.931, 1.154)	0.513
Brushing does not prevent gingivitis		
Incorrect answer	Ref.	
Correct answer	1.090 (0.978, 1.215)	0.119
Oral diseases may affect general health		
Incorrect answer	Ref.	
Correct answer	1.004 (0.909, 1.109)	0.932
*Oral health behaviours*		
Daily frequency of tooth brushing		
< 2 times/day	Ref.	
≥ 2 times/day	0.908 (0.823, 1.002)	0.056
Dental floss		
Non daily	Ref.	
Daily	0.868 (0.737, 1.022)	0.089
Smoking		
No	Ref.	
Yes	1.190 (0.966, 1.466)	0.102
Regular annual dental check-ups		
No	Ref.	
Yes	0.888 (0.790, 1.000)	0.049
Eating sugar and sweet food		
Once a day or less	Ref.	
More than once a day	0.913 (0.827, 1.008)	0.072
DMFT		0.712
0	Ref.	
0–1	1.038 (0.905, 1.191)	0.593
≥ 1	0.975 (0.872, 1.090)	0.652
Calculus level*		< 0.001
≤ 10%	Ref.	
> 10% and ≤ 30%	5.187 (4.623, 5.819)	< 0.001
> 30%	13.464 (10.798, 16.788)	< 0.001

*According to the proportion of calculus score, the calculus level was divided into ≤ 10%, > 10% and ≤ 30%, > 30%

The results of the multivariate logistic regression analysis are shown in Table 3. The association between gingivitis and age, being an only child, lack of regular annual dental check-up, and dental calculus level remained statistically significant after the inclusion of confounding variables. Compared to that in the 12-year-old group, the risk of gingivitis in the 14-year-old group increased by 21.6% (odds ratio [OR] = 1.216, 95% confidence interval [CI]: 1.044, 1.415). It should be noted that the percentage of dental calculus was an important risk factor for gingivitis in both unadjusted and adjusted models. After adjustment for gender and regional differences, compared with the group with at most 10% dental calculus, the risk of gingivitis in the groups with > 10–30% and above 30 % dental calculus increased by 5.490 (95% CI: 4.882, 6.175) and 15.803 (95% CI: 12.552, 19.896) times, respectively.

**Table 3:** The results of the multivariate logistic regression analysis

Variables	All		Urban		Rural		*P*_interaction_
	OR (95% CI)	*P*	OR (95% CI)	*P*	OR (95% CI)	*P*	
Age (year)							
12	Ref.						
13	1.030 (0.881, 1.201)	0.704	1.057 (0.854, 1.308)	0.609	1.011 (0.808, 1.265)	0.924	0.777
14	1.216 (1.044, 1.415)	0.012	1.075 (0.869, 1.329)	0.507	1.419 (1.139, 1.768)	0.002	0.075
15	0.699 (0.596, 0.821)	< 0.001	0.755 (0.605, 0.941)	0.013	0.646 (0.510, 0.818)	< 0.001	0.345
Gender							
Male	Ref.						
Female	0.968 (0.866, 1.081)	0.559	0.941 (0.806, 1.099)	0.443	0.986 (0.840, 1.157)	0.863	0.681
Only child							
No	Ref.						
Yes	1.236 (1.072, 1.425)	0.004	1.228 (1.037, 1.453)	0.017	1.333 (0.950, 1.870)	0.096	0.671
Father’s Education level							
≤ 9 years	Ref.						
10–12 years	0.897 (0.768, 1.047)	0.167	0.902 (0.732, 1.111)	0.331	0.859 (0.678, 1.087)	0.206	0.762
≥ 13 years	0.926 (0.726, 1.181)	0.537	0.896 (0.670, 1.199)	0.461	1.015 (0.630, 1.635)	0.950	0.662
Unclear	0.959 (0.757, 1.216)	0.729	0.797 (0.565, 1.123)	0.194	1.182 (0.843, 1.656)	0.332	0.108
Mother’s Education level							
≤ 9 years	Ref.						
10–12 years	1.022 (0.859, 1.216)	0.807	0.994 (0.794, 1.243)	0.956	1.053 (0.795, 1.396)	0.718	0.751
≥ 13 years	0.902 (0.693, 1.173)	0.440	0.868 (0.644, 1.172)	0.356	1.199 (0.643, 2.235)	0.567	0.360
Unclear	0.993 (0.785, 1.257)	0.956	1.002 (0.709, 1.414)	0.993	0.993 (0.715, 1.379)	0.968	0.973
Daily frequency of tooth brushing							
< 2 times/d	Ref.						
≥ 2 times/d	0.915 (0.817, 1.025)	0.124	0.871 (0.745, 1.018)	0.083	0.983 (0.831, 1.163)	0.844	0.301
Dental floss							
Non daily	Ref.						
Daily	0.936 (0.779, 1.125)	0.484	0.817 (0.648, 1.031)	0.088	1.246 (0.920, 1.689)	0.155	0.031
Regular annual dental check-up							
No	Ref.						
Yes	0.870 (0.763, 0.993)	0.038	0.826 (0.691, 0.986)	0.035	0.942 (0.774, 1.147)	0.552	0.328
Calculus level							
≤ 10%	Ref.						
> 10% and ≤ 30%	5.490 (4.882, 6.175)	< 0.001	4.556 (3.869, 5.365)	< 0.001	6.765 (5.699, 8.031)	< 0.001	0.001
> 30%	15.803 (12.552, 19.896)	< 0.001	15.413 (11.094, 21.413)	< 0.001	16.852 (12.161, 23.354)	< 0.001	0.706

When the children were stratified, we found that the association of gingivitis with these factors was inconsistent among the urban and rural areas. There was no statistical difference in the risk of gingivitis between 14-year-old children and 12-year-old children in urban areas (OR = 1.075, 95% CI: 0.869, 1.329), whereas the risk of gingivitis for 14-year-old children in rural areas was 1.419 (95% CI: 1.139, 1.768) times higher than that of 12-year-old children. Being an only child was inversely associated with risk of gingivitis in urban areas (OR = 1.228, 95% CI: 1.037, 1.453), but not in rural areas (OR = 1.333, 95% CI: 0.950, 1.870). Concomitantly, no associations were observed for the rural area group (OR = 0.942, 95% CI: 0.774, 1.147), although inverse associations with gingivitis were suggested with regular annual dental check-up for the urban area group (OR = 0.826, 95% CI: 0.691, 0.986). Moreover, compared with that of the low calculus rate group, the OR of moderate and high calculus groups in the urban population was 4.556 (95% CI: 3.869, 5.365) and 15.413 (95% CI: 11.094, 21.413), respectively, whereas in the rural population, it reached 6.765 (95% CI: 5.699, 8.031) and 16.852 (95% CI: 12.161, 23.354), respectively, which was higher than that in the urban population (*P*_interaction_ = 0.001).

## Discussion

A total of 29.6% of children aged 12–15 years were found to have gingivitis. It can be estimated that approximately 1.84 million children aged 12–15 years in Guangdong have gingivitis and may need medical intervention. However, we could not conclude that the prevalence of gingivitis among adolescents in Southern China is lower than that in other regions or countries since previous studies on the prevalence of gingivitis have used different examination protocols and criteria. Funieru et al. [10] and Amarasena and Ekanayake [26] reported that the prevalence of gingivitis was 91% in 10–17-year-old Bucharest school children and 86% in 15-year-old Sri Lankans, respectively. Both studies examined all the teeth present in the mouth and assessed the gingival status using the GI. The prevalence of gingivitis was calculated as a proportion of any GI mean score > 0 or 0.1, respectively. In previous studies, the CPI was applied to describe the gingival health condition, and a CPI-score of 1 was defined as gingival inflammation; therefore, the prevalence of gingivitis (or gingival inflammation) varied between 37.4 and 99% in children aged 11–15 years [11–13].

Due to the lack of a clear definition of gingivitis and differences in clinical gingival health assessment methods, it is difficult to compare the results of various epidemiological studies and infer a true difference in the prevalence of gingivitis. Because of financial and time limitations, we chose the method based on the WHO approach to detect gingival bleeding, which is particularly suitable for epidemiological field investigations. The majority of 15-year-olds in Southern China had intact periodontium. The rate of clinical AL ≥ 4 mm in 15-year-olds in this investigation was extremely low at 0.1%. Hence, for children aged 12–15 years, precision calibration probes (e.g. UNC-15 probe) are rarely needed to detect the PD or the AL in field investigation; thus, we chose the CPI probe as the measurement tool. Kingman et al. suggest that a full-mouth recording excluding wisdom teeth can be regarded as the gold standard for clinical examinations [27, 28]. In our survey, all teeth except the third molars were probed for assessing gingival health condition. Finally, the estimation of disease prevalence, evaluation of related risk factors, and disease surveillance require a disease definition. However, there has not been a generally accepted definition for gingivitis in the past. The joint EFP/AAP workshop in 2018 reported the definition of gingivitis, in which patients with intact periodontium would be diagnosed as having gingivitis if found with a BOP score ≥ 10%. Gingival bleeding is a sensitive indicator of gingival inflammation and gingival bleeding recording is recommended by the WHO for epidemiological surveys as it is economical and requires minimal/no technology [24]. Therefore, to better monitor the prevalence and severity of gingivitis among adolescents, we adopted gingival bleeding recording on all teeth in our study to define and grade gingivitis.

Bacterial biofilm is the primary cause of plaque-induced gingivitis. Dental calculus, when present as a calcified biofilm, is an ideal breeding environment for bacterial biofilm and is accepted as an important secondary etiological factor in the development and progression of periodontal diseases [18]. Similar to other studies, we also found that dental calculus was an important risk factor for gingivitis [26, 29, 30]. The level of the calculus was strongly associated with gingival status and showed a dose-response relationship in the adolescents. It is unsurprising that good oral hygiene habits such as flossing and brushing twice a day and regular dental check-up are protective factors against gingivitis. The removal of bacterial plaque and dental calculus is the most important objective of periodontal therapy and prevention. However, we found that students who held the ‘bleeding from brushing is normal’ and ‘brushing does not prevent gingivitis’ attitudes had a higher risk of gingivitis. This suggested that gingival bleeding was not generally accepted as a warning sign of gingivitis among these adolescents, and brushing alone did not improve bleeding in some adolescents with gingivitis, which needs further investigation.

Epidemiologic surveys reported that gingivitis is more prevalent in males than in females [31, 32]. Furuta et al. suggested that sex-based differences in gingivitis in young people could be explained by oral health behaviours and hygiene statuses because females had greater knowledge, a more positive attitude, a healthier lifestyle, and better oral health behaviours than males [17]. In contrast, Lock et al. reported that girls had a markedly higher prevalence of gingivitis than boys, especially among obese girls [33]. Differences between genders could be partly attributed to the regulatory effect of sex steroid hormones on gingival physiology. Increased levels of oestrogen and progesterone during pregnancy or puberty have been reported to result in increased gingival vascularity and inflammation [3]. Mealey et al. suggested that female sex steroid hormones may alter periodontal tissue responses to microbial plaque, and thus indirectly contribute to periodontal disease [34]. However, in our survey, gingivitis was only slightly more prevalent in males than in females in the univariate logistic regression analysis, but no significant gender differences were found for the prevalence of gingivitis in the multivariate logistic regression analysis.

Socioeconomic factors such as parental education, economic level of family, provision of dental care, and behaviours are combined factors in the development of gingivitis in adolescents. Most families in lower socioeconomic areas tend to have lower incomes, parents with lower levels of education, lack of dental services, and poorer oral hygiene. Several studies have reported a strong association between a low socioeconomic level and a high risk of periodontal diseases in children, higher prevalence of gingival bleeding, calculus, poorer oral hygiene, and AL of ≥ 3 mm [35–37]. In Southern China, the level of economy and consumption in rural areas is lower than that in urban areas. However, our study found no significant urban-to-rural differences in the prevalence of gingivitis among the 12–15-year-old groups. Nevertheless, when the adolescents were stratified by urban and rural areas, behaviours such as use of dental floss and regular annual dental check-up became protective factors of gingival health in urban children, but not in rural children. It was assumed that the rural children might lack access to good oral health care services that the urban children did not. Therefore, it should be emphasised that the oral health policy should pay more attention to children’s periodontal health and strengthen children’s oral health services. The primary focus of activities in periodontal care should be about health promotion and education, which might lead to improved oral hygiene. China’s one-child policy has been in place for around 60 years. Apart from rural children, most urban middle-class children aged 12–15 years are the only children in Guangdong. Surprisingly, it was found that the only child had a higher risk of gingivitis than the child with a sibling; this difference needs to be explored further.

## Conclusions

Dental calculus and oral health behaviour were important factors for the gingival health of children aged 12–15 years in Guangdong, Southern China. Maintaining gingival health in children requires promoting positive oral health behaviours and regular dental prophylaxis.

## Data Availability

The datasets generated during and analyzed during the current study are not publicly available due to privacy or ethical restrictions but are available from the corresponding author on reasonable request.

## Abbreviations

AL: Attachment loss
BOP: Bleeding on probing
CPI: Community Periodontal Index
CPITN: Community Periodontal Index of Treatment Needs
GI: Gingival index
OR: Odds ratio
PD: Periodontal pocket
WHO: World Health Organization
